# Demographic and socioeconomic characteristics of Canadian medical students: a cross-sectional study

**DOI:** 10.1186/s12909-020-02056-x

**Published:** 2020-05-12

**Authors:** Rishad Khan, Tavis Apramian, Joel Hosung Kang, Jeffrey Gustafson, Shannon Sibbald

**Affiliations:** 1 Schulich School of Medicine and Dentistry, Western University, London, Canada; 2 Centre for Education Research & Innovation, Schulich School of Medicine and Dentistry, Western University, London, Canada; 3 Faculty of Health Sciences, Western University, London, Canada; 4 Department of Family Medicine, Schulich School of Medicine and Dentistry, Western University, London, Canada; 5 Schulich Interfaculty Program in Public Health, Schulich School of Medicine and Dentistry, Western University, London, Canada

**Keywords:** Undergraduate medical education, Admissions, Socioeconomic status

## Abstract

**Background:**

While the importance of medical students’ demographic characteristics in influencing the scope and location of their future practice is recognized, these data are not systematically collected in Canada. This study aimed to characterize and compare the demographics of Canadian medical students with the Canadian population.

**Methods:**

Through an online survey, delivered in 2018, medical students at 14 English-speaking Canadian medical schools provided their age, sex, gender identity, ethnicity, educational background, and rurality of the area they grew up in. Respondents also provided information on parental income, occupation, and education as markers of socioeconomic status. Data were compared to the 2016 Canadian Census.

**Results:**

A total of 1388 students responded to the survey, representing a response rate of 16.6%. Most respondents identified as women (63.1%) and were born after 1989 (82.1%). Respondents were less likely, compared to the Canadian Census population, to identify as black (1.7% vs 6.4%) (*P* < 0.001) or Aboriginal (3.5% vs. 7.4%) (*P* < 0.001), and have grown up in a rural area (6.4% vs. 18.7%) (*P* < 0.001). Respondents had higher socioeconomic status, indicated by parental education (29.0% of respondents’ parents had a master’s or doctoral degree, compared to 6.6% of Canadians aged 45–64), occupation (59.7% of respondents’ parents were high-level managers or professionals, compared to 19.2% of Canadians aged 45–64), and income (62.9% of respondents grew up in households with income >$100,000/year, compared to 32.4% of Canadians). Assessment of non-response bias showed that our sample was representative of all students at English-speaking Canadian medical schools with respect to age, though a higher proportion of respondents were female. Additionally, there were no differences between early and late respondents with respect to ethnicity, rurality, and parental income, occupation, and education.

**Conclusions:**

Canadian medical students have different socioeconomic characteristics compared to the Canadian population. Collecting and analyzing these characteristics can inform evidence-based admissions policies.

## Background

Medical students differ from the general population with respect to socioeconomic status (SES), ethnicity, and rural background [1–3]. These differences may contribute to inequities in access to care, as many medical trainees go on to care for populations with whom they have shared life experiences and are comfortable serving [4–7]. The Association of Faculties of Medicine of Canada (AFMC) has called for medical schools to diversify their student population to more closely represent the Canadian population [8]. While several initiatives to respond to this call are underway, there is a lack of data on student demographics to inform future initiatives, support policy changes, and track progress [9].

In Canada, entry into medical school generally encompasses eligibility criteria, academic performance, application components such as essays and reference letters, and interviews [10]. Eligibility criteria can include a required number of years of undergraduate education and completion of the Medical College Admissions Test (MCAT). There are several distinctions between schools in Québec and those outside. Québec schools require a diploma or degree equivalent to a College studies diploma given by the Québec Ministry of Education, rather than an undergraduate degree [10]. Québec schools also do not require the MCAT. Outside of applications, Québec schools have seen a relatively modest rise in tuition compared to schools elsewhere. For example, tuition at the University of Toronto has risen from $3222/year in 1994 to $29,030/year in 2019. Conversely, tuition fees at the University of Montréal have risen from $2286 to $3601 for Quebec residents, and $11,193 for all other Canadians [1, 10]. Within this Canadian application system, equitable access to medical school may impact applicant pools, medical class composition, and future patient care.

Physicians who are part of visible minority populations backgrounds tend to treat traditionally underserved patients and serve in areas of physician shortage [11–18]. Students with low socioeconomic backgrounds and those who grew up in rural communities are more likely to serve communities with similar backgrounds and/or demographic characteristics [19–25]. The potential benefit to underserved communities has brought medical school admissions into the realm of social accountability. Indeed, Health Canada and the AFMC have highlighted the role of enhancing admissions processes to achieve the desired diversity in the physician workforce [26, 27]. Several Canadian medical schools have increased efforts to recruit underrepresented students, such as the Northern Ontario School of Medicine’s recruitment of students with aboriginal backgrounds [28] and the University of Calgary’s Pathways to Medicine Program, which aims to support the enrollment and success of future medical students from traditionally under-represented groups throughout Alberta [29]. Additionally, the University of Toronto recently developed a Black Student Application Program, with the goal of increasing and supporting Black medical student representation [30].

While some schools track applicant demographic characteristics, there has been no national characterization of the demographics of Canadian medical students since 2007 [1]. In the 2007 analysis, investigators found substantial disparities between medical students and the Canadian population with respect to socioeconomic status. Given the implications of these demographic disparities on access to care and the current shortage of physicians in Canada, it is important to systematically track such demographic data. In this study, we aimed to characterize the demographics of students at English-speaking Canadian medical schools through a nationally-administered survey, and to compare them to the Canadian population.

## Methods

We conducted a cross-sectional study on the demographics of students at English-speaking Canadian medical schools in 2018 through an online survey. We adapted the study methodology from previous studies on this topic [1, 2]. We coordinated the project with student leaders from the Canadian Federation of Medical Students (CFMS), which represents 14 of 17 Canadian medical schools. We excluded students from the other three Canadian medical schools, based in Quebec, for two reasons. First, previous studies have postulated that these students have distinctive demographic characteristics compared to medical students from English-speaking schools [1, 3], given their younger average age at matriculation and substantially lower tuition fees [31, 32]. Additionally, we did not have a reliable method of reaching these students as they are not represented by the CFMS.

### Survey design

Through our survey, we aimed to capture information on the following demographic characteristics: ethnicity, gender identity, sex assigned at birth, socioeconomic status, and rurality of the area respondents grew up in. Additional elements of the survey included questions regarding characteristics and behavior after entering medical school including: debt burden, preference of future specialty and practice location, and the perceived impact of demographic and financial factors on future practice. The results of those post-admission questions are not reported in this study. Instead, we focus on the demographics of students admitted to Canadian medical schools, with plans to publish further analysis of all study data. We hosted the survey on an online survey platform Simple Survey (OutSideSoft Solutions Inc., Quebec, Canada). The complete survey and explanations of questions is available as Additional file 1.

We used two previous surveys as starting points to improve content validity and allow for direct comparisons to other populations: The 2016 Canadian Census [33], and a previously validated survey addressing this research topic [2]. Most individual survey items were taken verbatim from the Canadian Census. For rurality, and parental occupation, we used classifications different from the census, which is detailed below in the section *Survey Content*.

We piloted the survey with 16 medical students from across Canada and subsequently altered wording for certain questions to improve clarity and applicability to the medical student population.

### Survey content

We collected data on respondents’ ages, year of medical school, and level of education prior to medical school. We also asked about ethnicity using the same terms used in the Canadian census: Aboriginal, Arab, Black, Chinese, Filipino, Korean, Japanese, Latin American, South Asian (e.g. Indian, Bangladeshi, Sri Lankan), Southeast Asian (e.g. Cambodian, Indonesian, Thai), West Asian (e.g. Iranian), and “other”. Participants could choose more than one ethnicity. We also asked students about the size of the community they grew up in, using the 2016 Statistics Canada Population Centre and Rural Area classification [34]. A rural area was defined as having a population of < 1000 people, small and medium population centres as having populations of 1000–99,999, and large urban population centres as having populations of ≥100,000 [34].

To compare participants’ socioeconomic status to the Canadian population, we asked about three commonly used and well-validated markers of socioeconomic status: parental income, occupation, and education level [35–38]. For parental income and education level, we used similar income brackets and diploma or degree classifications respectively as the 2016 census [33]. For parental education level, we used a modified version of the Pineo-Porter Occupational Scale as has been used by Dhalla et al. [2, 39].

### Survey delivery

We contacted medical students through class email lists. The emails included information on the purpose of the study, contact information for the research team, the nature of voluntary participation, and a link to the survey. No individual emails were collected, used, or stored at any point during the study. After the initial email, participants received three biweekly reminder emails. The CFMS also promoted the survey through their social media accounts (Facebook and Twitter), and student leaders at individual schools delivered class announcements. The survey was open for a total of 10 weeks in spring 2018 to ensure coverage of different examination and vacation schedules.

### Analysis

We imported questionnaire data directly from SimpleSurvey software into SPSS Version 24 (IBM, Armonk, NY). We removed participants who declined to complete the survey at the informed consent step, and surveys which were started but not answered. When two or more consecutive surveys had identical answers and the former survey(s) had fewer questions completed, we assumed that this was the same participant who accessed the survey more than once. In these cases, we only considered the final response.

We used descriptive statistics to summarize responses to all questions and chi-squared tests to detect differences in characteristics of survey respondents and the general Canadian population via the 2016 Comprehensive Census.

### Assessment of nonresponse bias

We performed post-hoc analyses to assess for nonresponse bias using two approaches [40]. First, we compared our data on age and sex to the 2017 Canadian Medical Education Statistics (CMES) report published by Association of Faculties of Medicine of Canada, a dataset which represents the entire Canadian medical student population [31]. For age, we compared our fourth-year respondents to graduating medical students in CMES, the only group for whom age was available. For sex, we compared results from our question “sex assigned at birth” to the listed sex of the CMES 2017 population from all years of medical school. We restricted the above analyses to students from English-speaking Canadian medical schools.

Second, we compared the first 100 respondents to the last 100 respondents, with the assumption that late respondents are more similar to nonrespondents [41]. We compared ethnicity, rurality, and parental income, education, and occupation between these groups.

Finally, to assess non-response bias based on a respondent’s year of medical school, we compared answers between first and fourth years. We undertook this analysis after observing substantial variability in response rates between respondents of different medical school years. For these two groups, we compared ethnicity, rurality, and parental income, education, and occupation.

## Results

A total of 1388 students from 14 Canadian medical schools responded to our survey. Based on the total population of students at English-speaking medical schools stated in the 2017 CMES report [31], we had a response rate of approximately 16.6%. The characteristics of Canadian medical students in the study group are described below in comparison with the Canadian Census. The findings are summarized in Table 1. There were 451 (32.5%) respondents from first year of medical school, 421 (30.3%) from second year, 295 (21.3%) from third year, and 221 (15.9%) from fourth year. With respect to respondents’ education prior to medical school, 33 (2.4%) attained doctorate degrees, 10 (0.7%) attained other professional degrees, 320 (23.1%) attained master’s degrees, 899 (64.7%) obtained bachelor’s degrees, and 126 (9.1%) had diplomas or degrees below a bachelor’s degree. Among all respondents, 155 (11.1%) spent more than 6 years in post-secondary education prior to medical school, 469 (33.8%) spent 5–6 years, 583 (42.0%) spent 4 years, and 181 (13.0%) spent fewer than 4 years. The descriptive statistics that follow use the Canadian Census as a comparator.

**Table 1:** Background characteristics of participants

Year of birth	No. participants (%), *N* = 1388
Before 1986	87 (6.3)
1986–1989	161 (11.6)
1990–1993	703 (50.6)
After 1994	437 (31.5)
**Gender identify**	
Woman	876 (63.1)
Man	498 (35.9)
Trans woman or trans man^a^	3 (0.2)
Genderqueer or gender non-confirming	8 (0.6)
Not stated	3 (0.2)
**Highest degree prior to medical school**	
Bachelors	899 (64.7)
Masters	320 (23.1)
Professional degree (e.g. dentistry, law)	10 (0.7)
Doctorate	33 (2.4)
Other	126 (9.1)

^a^These categories were combined prior to data analysis to avoid potentially identifying data

### Ethnicity

Ethnicities differed significantly between respondents and the general population (*P* < 0.001, χ^2^ = 169, dF = 5) (Table 2). Respondents from our survey were more likely to identify as South Asian (*P* < 0.001) and Chinese (*P* < 0.001), and less likely to identify as black (*P* < 0.001), Aboriginal (*P* < 0.001), and white (*P* < 0.001) when compared to the census population.

**Table 2:** Self-identified ethnic background of respondents and Canadians aged 15–34 ^a^

Self-identified ethnic background	No. (%) of students ^b^ (Total: 1388)	No. (%) of Canadians (Total: 8,808,300)
Aboriginal	49 (3.5)	653,055 (7.4)
Black	23 (1.7)	561,865 (6.4)
Chinese	156 (11.2)	541,475 (6.1)
South Asian	122 (8.8)	613,805 (7.0)
White	1008 (72.6)	7,762,260 (88.2)
Other visible minority	130 (9.4)	959,630 (10.9)

^a^ Based on 2016 Canadian Census data. For the purposes of comparison, we have characterized *White* as the following responses: non-Aboriginal North America, Europe, and Oceania

^b^ Respondents to both our survey and the census were able to select more than one self-identified ethnic background. The sum of all ethnic origin responses is greater than the total population of respondents due to the reporting of multiple self-identified ethnic backgrounds

### Rurality

A total of 1351 (97.3%) of our respondents answered a question about the size of the area they primarily grew up in. There were 864 (62.2%) who grew up in large urban centres, defined as a population of 100,000 or more, 398 (28.7%) who grew up in a small or medium-sized centre, defined as a population of 1000–99,999, and 89 (6.4%) who grew up in a rural area, defined as a population of less than 1000. In comparison, 59.6% of 2016 census respondents lived in a large urban centre, 21.7% in a small or medium-sized centre, and 18.7% in a rural area. The proportions differed significantly between survey respondents and the Canadian population, with Canadian medical students more likely to have grown up in urban centres (*P* < 0.001) and small or medium-sized centres (*P* < 0.001), and less likely to have grown up in a rural area (*P* < 0.001).

### Parental education and occupation

The education level differed significantly between respondents’ fathers and Canadian men aged 45–64 years old (*P* < 0.001, χ^2^ = 2130, dF = 3), and between respondents ‘mothers and Canadian women aged 45–64 years old (*P* < 0.001, χ^2^ = 1476, dF = 3) (Table 3). Respondents’ parents were more likely to have attained higher levels education, namely bachelor’s degrees (*P* < 0.001) and master’s or doctorate degrees (*P* < 0.001).

**Table 3:** Education level of respondents’ parents and Canadians aged 45–64 years ^a^

Occupation No. (%)	Respondents’ fathers ^b^	Male Canadians	Respondents’ mothers ^b^	Female Canadians
High school diploma or less	197 (14.2)	1,909,805 (39.7)	208 (15.0)	2,026,090 (40.2)
Diploma below bachelor’s	226 (16.3)	1,781,980 (37.1)	274 (19.8)	1,834,050 (36.4)
Bachelor’s degree	456 (32.9)	765,245 (15.9)	532 (38.6)	876,065 (17.4)
Master’s or doctorate degree	468 (33.7)	351,475 (7.3)	335 (24.2)	302,315 (6.0)

^**a**^ Based on 2016 Canadian census

^b^ Forty-one students did not provide their father’s occupation and 38 students did not provide their mother’s occupation

Additionally, our respondents’ parents had significantly different occupations compared to age-matched men (*P* < 0.001, χ^2^ = 1027, dF = 4) and women (*P* < 0.001, χ^2^ = 1306, dF = 4) (Table 4). Respondents’ fathers and mothers were more likely to be professionals or high-level managers (*P* < 0.001). Among respondents’ parents, 9.7% of fathers were physicians, compared to 5.8% of Canadian men aged 45–64, and 6.8% of mothers were physicians (*P* < 0.001), compared to 4.6% of Canadian women aged 45–64 (*P* < 0.001).

**Table 4:** Profession of respondents’ parents and working Canadians aged 45–64 years ^a^

Occupation No. (%)	Respondents’ fathers ^b^	Male Canadians	Respondents’ mothers ^b^	Female Canadians
Professional, high-level manager	892 (64.3)	693,270 (18.0)	764 (55.1)	730,520 (20.5)
Semiprofessional, technician, middle manager	90 (6.5)	803,385 (20.8)	83 (6.0)	642,975 (18.1)
Supervisor, foreperson	76 (5.5)	1,138,905 (29.5)	39 (2.8)	1,319,465 (37.1)
Skilled, semiskilled or unskilled labourer	257 (18.5)	1,163,395 (30.2)	275 (19.8)	808,060 (22.7)
Not applicable	34 (2.4)	58,110 (1.5)	186 (13.4)	58,315 (1.6)

^**a**^ Based on a modified Pineo-Porter Scale and the 2016 Canadian Census National Occupation Classification

^b^ Thirty-eight students did not provide their father’s occupation and 40 students did not provide their mother’s occupation

### Household income

Respondents from our survey had significantly different household incomes compared to the Canadian population (*P* < 0.001, χ^2^ = 618, dF = 4) (Table 5). Respondents were more likely to come from high-income households, with 62.9% of respondents indicating household income of greater than $100,000 CAD compared to 32.4% of the census population (*P* < 0.001).

**Table 5:** Income of respondents’ parental households and Canadian households ^a^

Survey income bracket *Canadian dollars*	No. (%) of students’ parental households ^b^	No. (%) Canadian households
< 20,000	33 (2.4)	1,369,630 (9.7)
20,000-39,999	69 (5.1)	2,351,595 (16.7)
40,000-59,999	134 (9.9)	2,271,780 (16.2)
60,000-99,999	267 (19.7)	3,517,155 (25.0)
> 100,000	851 (62.9)	4,561,920 (32.4)

^**a**^ Based on 2016 Canadian Census household income data

^b^ Thirty-four students did not respond to this question

### Assessing non-response Bias

We compared respondents in our survey to the entire population of students at English-speaking Canadian medical schools based of CMES 2017. We found no differences in age among graduating students. We did, however, find that students in our survey were more likely to have selected “Female” as the sex assigned at birth, compared to the CMES population (Additional file 2).

When comparing early to late respondents, defined as the first and last 100 respondents respectively, we found no differences with respect to ethnicity, rurality, and parental income, occupation, and education (Additional file 2).

When comparing first year respondents and fourth year respondents, we found no differences with respect to ethnicity, rurality, and parental income, occupation, and education.

## Discussion

We found several important differences between students from English-speaking medical schools in Canada and the general Canadian population. Medical students, compared to the census population, are more likely to have grown up in high-income households and have parents who are professionals with high levels of formal education. Medical students are less likely to be black, Aboriginal, and to have grown up in a rural setting. Our data add to numerous previous reports, dating back to the 1960s, of such disparities [1, 2, 42].

Accurately comparing our findings to earlier surveys conducted in 2001 and 2007 remains challenging due to our low response rate, changes in the broader Canadian population, and the capture of data from medical students from Quebec in previous studies [1, 2]. In our study, 62.9% of our respondents came from households earning more than $100,000 per year, compared to 46.7% in a 2007 survey and 36.5% in a 2001 survey [1]. Conversely, 7.5% of students in our survey came from households earning less than $40,000 per year, compared to 12.8% in 2007 and 17.6% in 2001 [1]. These income data, however, should be interpreted cautiously due to inflation and rising average income in Canada. In light of the low response rates and historical changes in income, further qualitative comparisons of the 2001 and 2007 data suggest that there may be increasing matriculation of students who are the children of highly-educated professionals, including physicians.

There may be several underlying reasons for this socioeconomic disparity. First, increasing tuition fees may affect enrollment patterns, as average first-year tuition fees at English-speaking medical schools in Canada have risen from $12,512 in 2007 to $18,594 in 2017 [31, 32]. A 2008 analysis of tuition deregulation in Ontario found that increasing tuition fees are associated with increased enrollment of students whose parents hold a graduate or professional degree [43]. Additionally, an increase in medical school tuition is associated with matriculation of fewer students from low-income families [3] and increasing socioeconomic status of enrolled students [44]. Conversely, schools with lower tuition fees are more likely to have students from low-income neighborhoods [1].

In addition to the potential impact of increasing tuition fees, increasing competition for a limited number of seats at medical schools may favor applicants with higher socioeconomic status [45]. Factors such as grade-point average and the MCAT are often weighted heavily for their perceived validity [45, 46]. While these measures have been shown to predict performance in medical school [47, 48], the advent of expensive test-preparation courses has commercialized the admissions process [49]. Furthermore, the emphasis on personal factors such as leadership, commitment to service, and volunteerism can create additional bias [45, 50]. Applicants with socioeconomic barriers may be unable to access experiences which emphasize these qualities or may be compelled to eschew such opportunities in favor of paid employment.

Encouragingly, many schools are attempting to make progress in this area. While only 3.5% of respondents in our survey were Aboriginal, this figure may improve. Recently, all 17 medical schools across Canada made a commitment to ensure matriculation of a minimum number of students from Aboriginal communities [51]. Additionally, many of our respondents grew up in small, medium-sized or rural communities, which may represent the results of recent efforts to recruit individuals from Aboriginal [26, 28] and rural communities [4].

### Limitations

Our study has several important limitations. First, we had a low response rate compared to previous studies of this kind. This biased our results towards more responses from female participants, as shown in our assessment of nonresponse bias. Our survey population, however, was representative in age, and that there were no differences between early and late respondents with respect to ethnicity and markers of socioeconomic status. Thus, a low response rate alone should not be considered as a marker of poor validity [40, 52, 53]. Second, our survey was voluntary and relied on self-reported data with no secondary verification, creating the opportunity for convenience, recall, and misclassification biases. We did, however, pledge anonymity and confidentiality to respondents and are not aware of any reason for them to systematically provide dishonest answers. Third, it remains possible that some participants accidentally responded more than once. However, once we removed suspected duplicate entries, as detailed in our Methods section, we had no two individual surveys with identical answers. Fourth, the generalizability of our results is limited as our survey was not sent to students from French-speaking medical schools, who are known to have differing demographics compared to their colleagues from English-speaking schools [1, 3]. Finally, we did not collect data on which individual school participants were from, which creates the possibility certain schools are over- or under-represented.

### Implications

We emphasize caution in the interpretation and generalization of our results, given the above limitations. Additionally, the relatively small samples of certain populations, such as the 23 respondents who identify as Black and the 10 respondents who attained a professional degree prior to medical school, make these particular subgroup comparisons challenging to interpret. Within these limitations, these data have several implications for medical education and health policy in Canada. Widening socioeconomic disparity between physicians-in-training and their future population may exacerbate inequities in access to care. A large body of evidence suggests that medical students from traditionally disadvantaged backgrounds, such as those who are part of visible minority populations [11–18] or have rural or low socioeconomic backgrounds [19–25], are more likely to practice in areas with physician shortage.

Inequities in medical school admission poses a ‘wicked’ political problem [54]. Addressing such inequities in the admissions process will take a large, coordinated effort. The first step in this effort, is the collection and dissemination of data on medical school applicants and matriculants. While student-initiated research in this domain, such as our survey, is a meaningful step, such efforts are sporadic and limited in scope. Indeed, our findings were substantially limited by the low response rate. Improving the quality of these data will require partnership between students, faculty, and funding bodies to systematically and continuously track educational outcomes and future practice locations of medical students from differing backgrounds [55]. Incorporating data on medical school applicants, in addition to matriculants, may further delineate the stages at which these inequities arise and persist.

Admittedly, simply collecting more data will not solve the problem of the socioeconomic gap between physicians and their patients. The availability of these data, however, can allow researchers, faculties of medicine, and governmental funding organizations from across the political spectrum to define the nature of the problem and adopt a more evidence-based approach to admissions policies.

## Conclusions

Through a cross-sectional survey conducted in 2018, we found that students at English-speaking Canadian medical schools have, on average, substantially higher socioeconomic status compared to the Canadian population. Compared to previous studies on this topic, the socioeconomic gap between medical students and the broader Canadian population appears to be widening. Addressing this complex issue will require a coordinated effort between students, medical schools and faculty, and funding bodies.

## Supplementary information

**Additional file 1.** Survey delivered to Canadian medical students.

**Additional file 2.** Supplementary data for non-response bias assessment.

## Data Availability

The following datasets used in the study are publicly available at the following links: a. Canadian Medical Education Statistics 2007: https://afmc.ca/sites/default/files/documents/en/Publications/CMES/Archives/CMES2007Vol29.pdf b. Canadian Medical Education Statistics 2017: https://afmc.ca/sites/default/files/CMES2017-Complete.pdf c. 2016 Canadian Census: https://www12.statcan.gc.ca/census-recensement/2016/dp-pd/index-eng.cfm The dataset of Canadian medical students from the 2018 survey is not available due to concerns regarding compromise of individual privacy.

## Abbreviations

*SES*: socioeconomic status
*AFMC*: Association of Faculties of Medicine of Canada
*CFMS*: Canadian Federation of Medical Students
*CMES*: Canadian Medical Education Statistics
*MCAT*: Medical College Admissions Test
