# Supplementary material for: Demographic and socioeconomic characteristics of Canadian medical students: a cross-sectional study
Source: BMC Med Educ. 2020 May 12;20:151. doi: 10.1186/s12909-020-02056-x (PMC7216658; doi:10.1186/s12909-020-02056-x)
Supplement: Supplementary file 1 — Additional file 1. Survey delivered to Canadian medical students. [file 12909_2020_2056_MOESM1_ESM.doc]

**Survey**

1. What year did you enter medical school? *[Drop-down box]*
   1. Before 2015
   2. 2015
   3. 2016
   4. 2017
2. What is your current gender identity?
   1. Male
   2. Female
   3. Trans male/Trans man
   4. Trans female/Trans woman
   5. Genderqueer/Gender non-conforming
   6. Different identity (please state):__________________
3. What sex were you assigned at birth, meaning on your original birth certificate?
   1. Male
   2. Female
4. In what year were you born? *[Drop-down box]*

- 1. Before 1985
  2. 1986
  3. 1987
  4. 1988
  5. 1989
  6. 1990
  7. 1991
  8. 1992
  9. 1993
  10. 1994
  11. 1995
  12. 1996
  13. 1997
  14. After 1997

*The following questions have been taken from Statistics Canada’s 2016 Census; We ask this question to be able to make comparisons to the Canadian Census data. Please respond as accurately as possible.*

1. Are you:

*****Please mark more than one if applicable*****

White

 Aboriginal (e.g., North American Indian, Metis, or Inuit)

######  Chinese

 South Asian (e.g., East Indian, Pakistani, Sri Lankan, etc.)

 Black

 Filipino

 Latin American

 Southeast Asian (e.g., Cambodian, Indonesian, Laotian, Vietnamese, Malaysian, etc.)

 Arab

 West Asian (e.g., Afghan, Iranian, etc.)

 Japanese

 Korean

 Other please specify _____________

1. Were you born in Canada? *[Radio buttons]*
   1. Yes
   2. No

If you answered 'Yes', please go to question 8.

1. Please indicate your status in Canada. *[Radio buttons plus textbox for other]*
   1. Canadian citizen
   2. Permanent resident (landed immigrant)
   3. Other _____________
2. How many years of post-secondary education did you complete before entering medical school? (Quebec students: Please do not include CEGEP) *[Drop down box]*
   1. 0
   2. 1
   3. 2
   4. 3
   5. 4
   6. 5
   7. 6
   8. 7
   9. 8
   10. 9
   11. 10
   12. >10
3. What degrees/diplomas did you complete prior to entering medical school? *****Select all that apply***.** *[Check boxes]*

######  None

######  Diplome d’étude collegial (CEGEP)

######  Bachelor’s

######  Master’s

######  Degree in dentistry, veterinary medicine, optometry or law

######  Doctorate

1. Please enter the first three digits of the postal code where you lived in your final year of high school. *If you lived at a boarding school, please enter the postal code of your permanent residence (e.g. parent’s home) during that year. If you did not live in Canada, please skip this question.* *[Text box]*
2. Which type of place did you primarily grown up in?
   1. Village (population <1000)
   2. Town (population 1000-100,000)
   3. City (population >100,000)
3. What is your best estimate of the total income, before taxes and deductions, of your parental household in the last 12 months? *If your parents are divorced or separated, please enter the household income of the parent who supports or supported you most. [Drop down box]*
   1. Less than $20,000
   2. $20,000 - $39,999
   3. $40,000 - $59,999
   4. $60,000 - $79,999
   5. $80,000 - $99,999
   6. $100,000 - $119,999
   7. $120,000 - $139,999
   8. $140,000 - $159,999
   9. $160,000 - $179,999
   10. $180,000 - $219,999
   11. $220,000 - $259,999
   12. $260,000 - $299,999
   13. More than $300,000
4. What certificates, diplomas, or degrees did your parents obtain? *[Check boxes]*

**Please check all that apply**

|  | Father | Mother |
| --- | --- | --- |
| None |  |  |
| High school graduate or equivalent |  |  |
| Trades certificate or diploma |  |  |
| Other non-university certificate or diploma (community college, CEGEP, technical institute, etc.) |  |  |
| University certificate or diploma below bachelor level |  |  |
| Bachelor's degree(s) (e.g., B.A., B.Sc., LL.B.) |  |  |
| University certificate or diploma above bachelor level |  |  |
| Master's degree(s) (e.g., M.A., M.Sc., M.Ed.) |  |  |
| Degree in medicine, dentistry, veterinary medicine or optometry (M.D., D.D.S., D.M.D., D.V.M., O.D.) |  |  |
| Earned doctorate (e.g., Ph.D., D.Sc., D.Ed.) |  |  |

1. Please enter the category that best applies to your parents’ occupations. If your parents are retired or deceased, please provide the main occupation while working. *[Radio buttons]*

|  | Father | Mother |
| --- | --- | --- |
| Farmer or farm labourer, unskilled or semi-skilled manual, | O | O |
| clerical, sales or service |  |  |
| Skilled craft, trade, clerical, sales or service | O | O |
| Foreman/forewoman, supervisor, or middle manager | O | O |
| Technician or semi-professional | O | O |
| High-level management | O | O |
| Employed professional | O | O |
| Self-employed professional | O | O |
| Not applicable | O | O |

1. Please select one of the following. *[Radio buttons]*

O Neither of my parents are medical doctors.

O Both my parents are medical doctors.

O My mother is a medical doctor.

O My father is a medical doctor.

1. If you had to choose your medical practice today, which of the following would you choose? *[Radio buttons, text box]*

O University affiliated; basic science teaching and/or research

O University affiliated practice; clinical teaching, research and/or patient care

O Non-academic clinical practice

O Governmental agency (e.g. armed forces, medical health officer, Health Canada, etc.)

O Other (please specify) ________________

1. Are you presently enrolled in a ‘return of service’ program (i.e., a program where you have committed to certain practice restrictions [location, specialty, employer, etc.] in return for financial compensation during medical school or residency)? *[Radio buttons]*

O Yes

O No

1. How much did financial considerations affect your choice of medical school? *[Radio buttons]*

Not at all Most important factor

O O O O O

1. How much do financial considerations affect your choice of specialty? *[Radio buttons]*

Not at all Most important factor

O O O O O

1. How much do financial considerations affect your choice of future practice location? *[Radio buttons]*

Not at all Most important factor

O O O O O

1. How much did where you grew up affect your choice of medical school? *[Radio buttons]*

Not at all Most important factor

O O O O O

1. How much does where you grew up affect your choice of specialty? *[Radio buttons]*

Not at all Most important factor

O O O O O

1. How much does where you grew up affect your choice of future practice location? *[Radio buttons]*

Not at all Most important factor

O O O O O

1. How much do you anticipate spending on *non-tuition* expenses (e.g., rent, books, food, clothing, transportation, entertainment, etc.) 2017? *[Text box]*

$ ________

1. What activities did you engage in this past summer *for at least one week*? Select all that apply. *[Check boxes]*

 Required university educational activities

 Preparing for licensing examinations

 Informal clinical electives (unpaid)

 Rural clinical elective for which you were financially compensated

 Unpaid research

 Paid research

 Other paid employment

 Volunteer work

 Travel

1. Have you sought employment (e.g., a part-time job) during the current academic year? *[Radio buttons]*

O Yes O No

1. Do you currently have any debt (e.g., bank loans, government loans, family loans, etc.)? *[Radio buttons]*

O Yes O No

If you answered 'no', go to question __. If 'yes', continue with the next question.

1. How much total debt (e.g., bank loans, government loans, family loans, etc.) did you have upon entering medical school? (Please enter your best estimate if you are not sure of the exact amount) *[Text box]*

$ ________

1. How much total debt did you have as of August 1 of the current academic year (2017-2018)? (Please enter your best estimate if you are not sure of the exact amount) *[Text boxes]*

Bank/other financial services: $ ________

Provincial/federal government loans: $ ________

Family loans: $ ________

Credit cards (include only balance upon which you pay interest): $ ________

1. How much total debt (e.g., bank loans, government loans, family loans, etc.) do you expect to have at graduation from medical school? *[Text box]*

$ ­________

1. How much total financial assistance do you expect to receive in the form of *non-repayable* gifts or grants this academic year (2016-2017)? *[Text box]*

Government grants: $ ________

University or faculty grants or bursaries: $ ________

###### Scholarships and awards: $ ________

Family contributions: $ ________

Other grants or gifts: $ ________

1. Which of the following best describes your financial situation? *[Radio buttons]*

O Not stressful

O Minimally stressful (I think about it occasionally)

O Fairly stressful (I think about it each day and have to consciously mind what I buy)

O Very stressful (I worry that I am heading for major difficulties in the future)

O Extremely stressful (I worry about it constantly – this is my main source of stress)

Thank you for completing this survey. If you have any comments about this survey or any issues raised, please use the space provided below.

**Appendix B: Question Explanations**

Question 1: Each student will be given a unique identifier and asked to enter it with their responses, for two reasons. First, the unique identifier will allow us to contact non-responders and encourage them to complete the survey. Second, the unique identifier will allow us to track changes on a year-to-year basis.

Questions 2-4: No explanation necessary.

Question 5: This question is essentially identical to the one posed in the Canadian census, and will allow us to compare the Canadian medical student population to the national population.

Question 6: This question will also allow us to compare medical students to the national population.

Question 7: This question is asked so that foreign students can be excluded from the analysis.

Question 8-9: No explanation necessary.

Question 10: The first three digits of a postal code correspond to a Forward Sortation Area, for which Statistics Canada publishes detailed information.

Question 11: This question is similar to one posed in the Canadian census, and will allow us to compare the Canadian medical student population to the national population.

Question 12: This question is essentially identical to one posed in the Canadian census, and will allow us to compare the Canadian medical student population to the national population.

Questions 13: The student selects from a modified Pineo-Porter classification, thus eliminating the need for coding. These job classifications are correlated with socioeconomic status.

Question 14: No explanation necessary.

Question 15: This question is loosely based on one asked by the American Association of Medical Colleges in its Graduation Questionnaire.

Question 16-30: No explanation necessary.
