# Supplementary material for: Demographic and socioeconomic characteristics of Canadian medical students: a cross-sectional study
Source: BMC Med Educ. 2020 May 12;20:151. doi: 10.1186/s12909-020-02056-x (PMC7216658; doi:10.1186/s12909-020-02056-x)
Supplement: Supplementary file 2 — Additional file 2. Supplementary data for non-response bias assessment. [file 12909_2020_2056_MOESM2_ESM.docx]

**Supplementary Table 1:** Comparison of age between graduating medical students in survey population and student population at English-speaking Canadian medical schools.

| Age | No. (%) of survey respondents  (Total: 221) | No. (%) of Canadian Medical Students ^a^ (Total: 2122) |
| --- | --- | --- |
| 24-27 | 134 (60.6) | 1296 (61.1) |
| 28-31 | 62 (28.1) | 601 (28.3) |
| 32+ | 25 (11.3) | 225 (10.6) |

^a^ Data extracted from the Canadian Medical Education Statistics 2017 document, which includes data collected annually by the Association for Faculties of Medicine of Canada on all medical students

**Supplementary Table 2:** Comparison of sex assigned at birth of survey population and sex of entire student population at English-speaking Canadian medical schools.

| Sex | No. (%) of survey respondents ^a^  (Total: 1388) ^b^ | No. (%) of Canadian Medical Students ^c^  (Total: 8359) |
| --- | --- | --- |
| Woman | 885 (63.8) | 4381 (52.4) |
| Man | 501 (36.1) | 3978 (47.6) |

^a^ Sex assigned at birth, as answered by survey participants

^b^ Two respondents did not answer this question

^c^ Data extracted from the Canadian Medical Education Statistics 2017 document, which includes data collected annually by the Association for Faculties of Medicine of Canada on all medical students

**Supplementary Table 3:** Comparison of self-identified ethnic background between early and late responders in our survey

| Self-identified ethnic background | No. of early responders ^a^  (Total: 100) ^c^ | No. of late responders ^b^  (Total: 100) ^c^ |
| --- | --- | --- |
| Aboriginal | 1 | 1 |
| Black | 2 | 3 |
| Chinese | 16 | 8 |
| South Asian | 12 | 14 |
| White | 66 | 64 |
| Other visible minority | 11 | 13 |

^a^ Early responders are defined as the first 100 participants to respond to the survey

^b^ Late responders are defined as the last 100 participants to respond to the survey

^c^ Respondents to our survey were able to select more than one self-identified ethnic background. The sum of all ethnic origin responses is greater than the total population of respondents due to the reporting of multiple self-identified ethnic backgrounds.

**Supplementary Table 4:** Comparison of size of hometown as an index of rurality between early and late responders in our survey

| Size of hometown | No. of early responders ^a^  (Total: 100) ^c^ | No. of late responders ^b^  (Total: 100) ^c^ |
| --- | --- | --- |
| <1000 | 6 | 5 |
| 1000-100,000 | 24 | 24 |
| >100,000 | 69 | 70 |

^a^ Early responders are defined as the first 100 participants to respond to the survey

^b^ Late responders are defined as the last 100 participants to respond to the survey

^c^ One early responder and one late responder did not answer the question about the size of the place they primarily grew up in

**Supplementary Table 5:** Comparison of parental income, education, and occupation between early and late responders in our survey

| Characteristic | No. of early responders ^a^  (Total: 100) | | No. of late responders ^b^  (Total: 100) ^c^ | |
| --- | --- | --- | --- | --- |
| **Income bracket (Canadian dollars)** |  | |  | |
| <40,000 | 7 | | 8 | |
| 40,000-99,999 | 32 | | 26 | |
| 100,000-219,999 | 40 | | 45 | |
| >220,000 | 21 | | 19 | |
| **Parental Education** | Respondents’ fathers ^d^ | Respondents’ mothers ^d^ | Respondents’ fathers ^e^ | Respondents’ mothers ^e^ |
| High school diploma or less | 14 | 16 | 14 | 14 |
| Diploma below bachelor’s | 15 | 17 | 9 | 14 |
| Bachelor’s degree | 31 | 39 | 29 | 34 |
| Master’s or doctorate degree | 38 | 25 | 46 | 35 |
| **Parental occupation** ^f^ | Respondents’ fathers ^g^ | Respondents’ mothers ^g^ | Respondents’ fathers ^h^ | Respondents’ mothers ^h^ |
| Professional, high-level manager | 62 | 50 | 70 | 54 |
| Semiprofessional, technician, middle manager | 5 | 6 | 5 | 5 |
| Supervisor, foreperson | 10 | 4 | 2 | 1 |
| Skilled, semiskilled or unskilled labourer | 20 | 17 | 18 | 21 |
| Not applicable | 2 | 22 | 3 | 17 |

^a^ Early responders are defined as the first 100 participants to respond to the survey

^b^ Late responders are defined as the last 100 participants to respond to the survey

^c^ Three late responders did not answer this question

^d^ Two early responders did not provide their father’s education and three early responders did not provide their mother’s education

^e^ Two late responders did not provide their father’s education and three late responders did not provide their mother’s education

^f^ Based on a modified Pineo-Porter Scale and the 2016 Canadian Census National Occupation Classification.

^g^ One early responder did not provide their father’s occupation and one early responder did not provide their mother’s occupation one and one

^h^ Two late responders did not provide their father’s occupation and two late responders did not provide their mother’s occupation

**Supplementary Table 6:** Comparison of self-identified ethnic background between first-year and fourth-year responders in our survey

| Self-identified ethnic background | No. of first-year responders  (Total: 451) ^a^ | No. of fourth year responders  (Total: 221) ^a^ |
| --- | --- | --- |
| Aboriginal | 13 | 11 |
| Black | 7 | 2 |
| Chinese | 49 | 24 |
| South Asian | 37 | 12 |
| White | 325 | 173 |
| Other visible minority | 44 | 17 |

^a^ Respondents to our survey were able to select more than one self-identified ethnic background. The sum of all ethnic origin responses is greater than the total population of respondents due to the reporting of multiple self-identified ethnic backgrounds.

**Supplementary Table 7:** Comparison of size of hometown as an index of rurality between first-year and fourth-year responders in our survey

| Size of hometown | No. of first year responders  (Total: 451) ^c^ | No. of fourth year responders  (Total: 221) ^c^ |
| --- | --- | --- |
| <1000 | 27 | 14 |
| 1000-100,000 | 132 | 69 |
| >100,000 | 279 | 136 |

^c^ Sixteen first-year responders and two fourth-year responders did not answer the question about the size of the place they primarily grew up in

**Supplementary Table 8:** Comparison of parental income, education, and occupation between first-year and fourth-year responders in our survey

| Characteristic | No. of first year responders  (Total: 451) ^a^ | | No. of fourth year responders  (Total: 221) ^a^ | |
| --- | --- | --- | --- | --- |
| **Income bracket (Canadian dollars)** |  | |  | |
| <40,000 | 27 | | 21 | |
| 40,000-99,999 | 145 | | 89 | |
| 100,000-219,999 | 169 | | 63 | |
| >220,000 | 109 | | 47 | |
| **Parental Education** | Respondents’ fathers ^b^ | Respondents’ mothers ^b^ | Respondents’ fathers ^c^ | Respondents’ mothers ^c^ |
| High school diploma or less | 50 | 64 | 31 | 35 |
| Diploma below bachelor’s | 77 | 87 | 43 | 42 |
| Bachelor’s degree | 159 | 170 | 67 | 93 |
| Master’s or doctorate degree | 151 | 118 | 77 | 47 |
| **Parental occupation** ^d^ | Respondents’ fathers ^e^ | Respondents’ mothers ^e^ | Respondents’ fathers ^f^ | Respondents’ mothers ^f^ |
| Professional, high-level manager | 301 | 244 | 141 | 116 |
| Semiprofessional, technician, middle manager | 25 | 27 | 17 | 14 |
| Supervisor, foreperson | 26 | 14 | 10 | 6 |
| Skilled, semiskilled or unskilled labourer | 78 | 86 | 42 | 49 |
| Not applicable | 7 | 66 | 9 | 34 |

^a^ One first-year responder and one fourth-year responder did not provide parental income

^b^ Fourteen first-year responders did not provide their father’s education and twelve did not provide their mother’s education

^c^ Three fourth-year responders did not provide their father’s education and four did not provide their mother’s education

^d^ Based on a modified Pineo-Porter Scale and the 2016 Canadian Census National Occupation Classification.

^e^ Fourteen first-year responders did not provide their father’s occupation and fourteen did not provide their mother’s occupation

^f^ Two fourth-year responders did not provide their father’s occupation and two did not provide their mother’s occupation
